# Two Novel *KRIT1* and *CCM2* Mutations in Patients Affected by Cerebral Cavernous Malformations: New Information on *CCM2* Penetrance

**DOI:** 10.3389/fneur.2018.00953

**Published:** 2018-11-14

**Authors:** Concetta Scimone, Luigi Donato, Zoe Katsarou, Sevasti Bostantjopoulou, Rosalia D'Angelo, Antonina Sidoti

**Affiliations:** ^1^Department of Biomedical and Dental Sciences and Morphological and Functional Images, University of Messina, Messina, Italy; ^2^Department of Vanguard Medicine and Therapies, Biomolecular Strategies and Neuroscience, I.E.ME.S.T., Palermo, Italy; ^3^Department of Chemical, Biological, Pharmaceutical and Environmental Sciences, University of Messina, Messina, Italy; ^4^Department of Neurology, Hippokration General Hospital, Thessaloniki, Greece; ^5^3rd University Department of Neurology, Thessaloniki, Greece

**Keywords:** CCM, splicing variants, CCM2 penetrance, pathogenesis, CCM genotype-phenotype correlation

## Abstract

Wide comprehension of genetic features of cerebral cavernous malformations (CCM) represents the starting point to better manage patients and risk rating in relatives. The causative mutations spectrum is constantly growing. *KRIT1, CCM2*, and *PDCD10* are the three loci to date linked to familial CCM development, although germline mutations have also been detected in patients affected by sporadic forms. In this context, the main challenge is to draw up criteria to formulate genotype-phenotype correlations. Clearly, genetic factors determining incomplete penetrance of CCM need to be identified. Here, we report two novel intronic variants probably affecting splicing. Molecular screening of CCM genes was performed on DNA purified by peripheral blood. Coding exons and intron-exon boundaries were sequenced by the Sanger method. The first was detected in a sporadic patient and involves *KRIT1*. The second affects *CCM2* and it is harbored by a woman with familial CCM. Interestingly, molecular analysis extended to both healthy and ill relatives allowed to estimate, for the first time, a penetrance for *CCM2* lower than 100%, as to date reported. Moreover, heterogeneity of clinical manifestations among those affected carrying the same genotype further confirms involvement of modifier factors in CCM development.

## Introduction

Cerebral cavernous malformations (CCM, OMIM #116860) are vascular lesions affecting brain capillaries. Lesions appear like mulberries, due to the enlarged, tangled vessels which form them. The single layer of endothelial cells is really inhomogeneous as a result of pericyte absence and defective tight and adherens junctions (1) with consequent impairment of the blood-brain barrier. This condition leads to an increased bleeding risk of lesions. Intracerebral hemorrhage (ICH), together with seizures, are the main clinical manifestations appearing in up to 70% of symptomatic patients. Other symptoms include headache, focal neurological deficits, vertigo, and paresis.

However, only about 75% of affected individuals clinically manifest, with no gender predominance (2). CCM arise mainly in the supratentorial region, however, a more severe prognosis is associated to lesions affecting brainstem and pons. Extra-neurological localization was also observed, and others involved organs include kidney, liver, skin (3). Neuro-radiological diagnosis of CCM is performed by magnetic resonance imaging (MRI) with T-2 gradient-echo and susceptibility-weighted imaging. Heterogeneity of clinical manifestations and the age of symptoms onset are linked to incomplete penetrance and variable expressivity of the disease. Due to these conditions, worldwide incidence of CCM is underestimated and ranges between 0.4 and 0.8% (4). Lesion numbers are also quite variable and related to genetic etiology. Usually, single cavernomas are detected in patients affected by sporadic forms while multiple lesions are frequent in familial forms. Sporadic CCM arises mostly from the third decade of life on, due to altered expression of genes involved in regulation of the angiogenetic process, following hypoxia, inflammation, or oxidative stress conditions. Patients affected by familial forms, instead, often show multiple lesions already in childhood, as a consequence of mutations at the three different loci *KRIT1/CCM1* (Entrez Gene: 889; 7q11.2-21), *MGC4607/CCM2* (Entrez Gene: 83605; 7p13), and *PDCD10/CCM3* (Entrez Gene: 11235; 3q26.1). Clinical penetrance is estimated to be about 88% for *KRIT1*, 100% for *CCM2*, and 63% for *PDCD10* (5). Moreover, mutations at *PDCD10* seem to be linked to a more severe phenotype characterized by recurrent ICH (6). Three CCM genes encode for the proteins Krev interaction trapped 1 (krit1), malcavernin and programmed cell death protein 10 (pdcd10), involved in maintaining cell-cell junctions and cell-extra cellular matrix (ECM) adhesion, in the regulation of apoptosis/proliferation switch of endothelial cells and in response to oxidative damage (7). In particular, at cell–cell adherens junctions, the three CCM proteins interact forming a ternary complex in which malcavernin acts as a bridge between krit1 and pdcd10. Therefore, loss of function mutations causes a disassembly of the complex resulting in an increased migratory capacity and in a gain of mesenchymal phenotype of endothelial cells due to perturbation of β-catenin/TGF-β/BMPs signaling (8).

To date, more than 300 mutations at the three CCM loci are reported in the public version of the Human Gene Mutation Database (HGMD®) as disease causing mutations. Of these, 15 and 12 impair splicing of *KRIT1* and *CCM2* transcripts, respectively. Here, we report two novel intronic variants that affect *KRIT1* and *CCM2* genes, probably leading to aberrant splicing processes.

## Materials and methods

### Case descriptions

**Figure 1:**
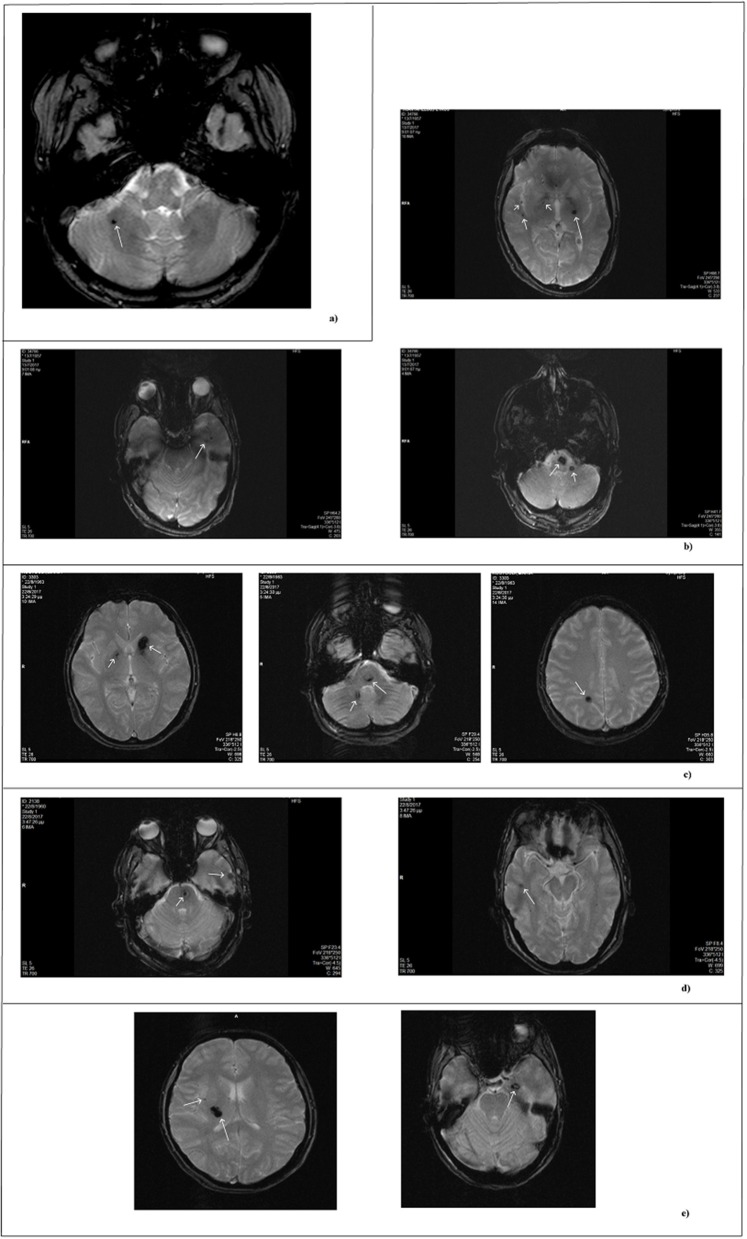
MRI scan of patients. MRI images obtained by T2- weighted gradient echo sequences. Each panel refers to a single patient. **(a)** Single lesion detected in the sporadic patient. **(b–e)** Lesions of affected family members. In reference to Figure 2: **(b)** II-1; **(c)** II-3; **(d)** II-4; **(e)** III-13.

The study involves two patients. The first is a 57-year-old Italian man who accidentally discovered to be affected, being asymptomatic. The single lesion at the right cerebellar hemisphere was diagnosed by an MRI exam performed after an accident (Figure 1a). No evidence of suspected affected relatives was reported by the patient and, therefore, he was classified as sporadic.

**Figure 2:**
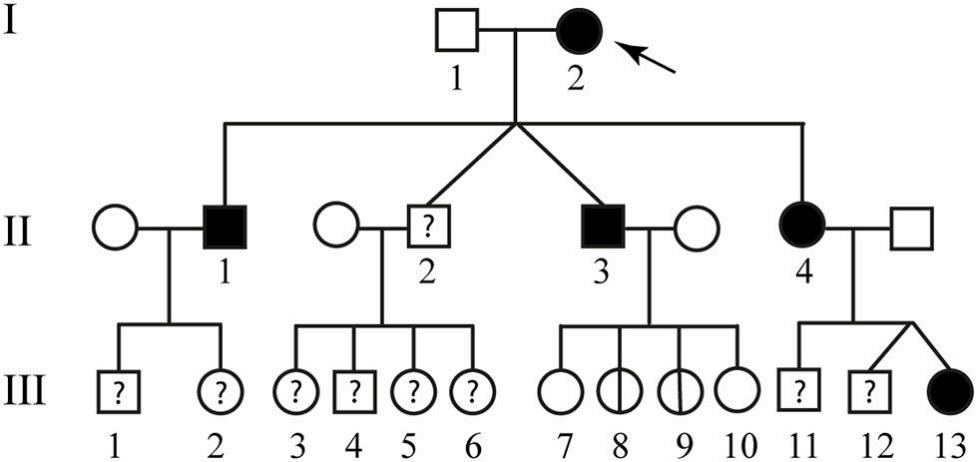
Familial pedigree of case report 2. The arrow indicates the proband. Healthy members are indicated by empty symbols. Empty circles vertically crossed refer to healthy carrier mutation. Affected ones are represented by black filled. By question mark are indicated consanguineous of which no genotype or phenotype data are available.

The second patient is an 84 year-old woman affected by hereditary CCM (Figure 2; I-2) diagnosed about 20 years ago following recurrent epileptic seizures. No MRI scan images are available; however, from medical reports read, the presence of four cavernous lesions emerged and, particularly, two at the pontine region and two at the right parietal lobe. The woman could not provide anamnesis information on her parents. The study was also extended to other members of the family and, particularly, to three of her sons and five grandchildren. Three of her sons, as reported in Figure 2, are affected. The first born is a 60-year-old man (Figure 2; II-1) who suffers from global transient amnesia without neurological deficits. MRI highlighted at least seven lesions, distributed among brainstem and both cerebellar hemispheres (Figure 1b). Diagnosis in one of the two second-born (a twin) Figure 2; II-3) was performed subsequently to familial history. Despite MRI showing the presence of four CCM lesions at brainstem (Figure 1c) the 58-year-old patient is, to date, asymptomatic. No information is available about his twin. The younger daughter (Figure 2; II-4) is 48 years old and manifests recurrent headaches, right hemiparesis, and hemi-hyperesthesia. These symptoms are due to five CCM lesions affecting brainstem and both cerebral hemispheres (Figure 1d). Regarding the five grandchildren, MRI was positive only for one of them (Figure 2; III-13) who, however, is asymptomatic. In detail, she carries three lesions, two in the area of the posterior limb of the right internal capsule and one in the left temporal lobe (Figure 1e). The patients involved in the study were fully informed and informed consent was obtained in accordance with the Declaration of Helsinki. Written informed consent for the publication of this study was obtained from the participants. The study was approved by our local Ethical Committee A.O.U. G. Martino Messina.

### Patient genotyping

Molecular screening of CCM genes was performed on DNA purified by peripheral blood. Coding exons and intron-exon boundaries were screened using primer pairs designed according to the *KRIT1, CCM2*, and *PDCD10* published nucleotide sequences of GenBank (accession numbers NG_012964.1, NG_016295.1, and NG_008158.1, respectively) and are available upon request. The sequence variations detected were described according to the recommendations by the Human Genome Variation Society, having the A of the ATG translation starting codon as +1 at the cDNA level. Amplified products were sequenced by Sanger method using the BigDye Terminator v3.1 chemistry (Applied Biosystems®, Thermofisher Scientific) and run on a 3500 Genetic Analyser (Applied Biosystems®, Thermofisher Scientific). Raw data analysis was performed by Applied Biosystems Sequencing Analysis Software® v6.0.

### Multiplex ligation-dependent probe amplification (MLPA) assay

In order to exclude the presence of large genomic rearrangements, MLPA was performed according to the manufacturer's instructions using two MLPA kits (SALSA MLPA Kits, P130 and P131 CCM, MRC-Holland). For visual inspection, peak heights were compared between the sample and controls to find any alteration in relative peak heights within the test sample. For the normalized peak-area calculations, each peak area was normalized by dividing the individual peak area by the total peak area of all peaks for that sample.

### Characterization of novel variants and of mutated proteins features by coding sequences study

Two novel intronic variants were detected by DNA direct sequencing in *KRIT1* and *CCM2* genes. To evaluate any splicing impairment, the transcripts of both genes were retro-transcribed, amplified, and sequenced. Total RNA was purified by whole peripheral blood using TRIzol® reagent (Thermo Fisher Scientific), following manufacturer protocol. Selective retro-transcription of mRNA was performed using GeneAmp RNA PCR Core Kit® (Thermo Fisher Scientific), applying the two-step protocol. Retro-transcription was followed by amplification of the partial *KRIT1* and *CCM2* coding sequences. In particular, the region between exons 13–15 was sequenced for *KRIT1*, using the primer pairs: 5′-GAA ATT CCT ACT TAT GGA GCA GC-3′ and 3′-GCA TTA ACT GTC CAT TTA GCT TCA-5′. Obtained amplicon has a length of 301 bp. Regarding *CCM2*, primer pairs 5′-ACT TTC TGC TTC CCT GAA TCT GT-3′ and 3′-AGT CCT GGT CCA TGC TGC AGC-5′ were used and a fragment of 529 bp between exons 8–10 was amplified. Detailed protocols are available upon request.

### Allele frequency estimation

Frequency in the population of these two novel variants was estimated by DNA direct sequencing and by restriction fragment length polymorphism (PCR-RFLP) analysis, performed on two different 150 members of healthy control groups, recruited from Italian and Greek populations. The groups were heterogeneous for age and sex.

In particular, the novel *KRIT1* variant does not affect any restriction site therefore it was screened by direct sequencing. Regarding *CCM2*, the novel variant leads to loss of a restriction site for PstI. The wild-type 499 base pairs (bp) genomic fragment has two restriction sites for PstI and digestion produces three fragments of 153, 259, and 87 bp. In the presence of the variant, the first restriction site is lost resulting in formation of only two fragments of 412 and 87 bp (not shown). Five PstI units were used for overnight digestion of 0.5 μg of DNA, incubating the reaction at 37°C. Controls consisted of two Italian and Greek groups, each made up of 150 healthy, unrelated, randomly selected subjects. The Italian group was made up of 72 males and 78 females, average age 41.8. The Greek group was formed by 77 males and 73 females, average age 46.3. All controls were recruited after having undergone MRI for non-specific reasons, such as headache, dizziness, or mental confusion and resulted negative.

### *In-silico* prediction of structural alterations and loss of interaction

As subsequently reported, only the *CCM2* variant affects splicing. Comparison between wild-type and mutated secondary structures was performed by “Structure Prediction” module of RaptorX tool (9), giving as input native and mutated malcavernin amino acid sequences in FASTA format.

## Results

### Patient genotyping

**Figure 3:**
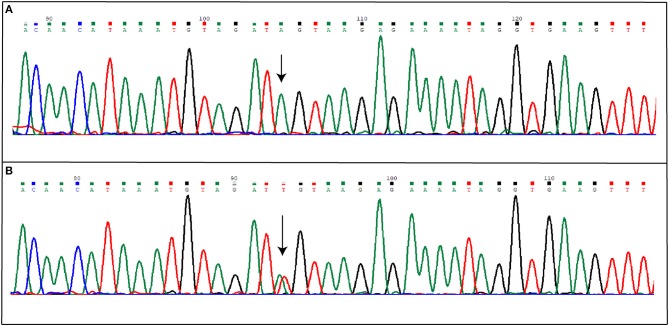
Novel IVS15-66A>T mutation detected in KRIT1 gene. The figure shows both wild-type **(A)** and mutated **(B)** electropherograms. The heterozygous substitution is indicated by the arrow.

Direct sequencing of coding regions and of intron-exon boundaries of the three CCM genes allowed to detect two novel intronic variants that may affect the splicing process. Specifically, the first patient affected by the sporadic form of CCM carries the nucleotide substitution IVS15-66A>T(c.2026-66A>T), located in the intron between coding exon 14 and 15 (10), affecting the adenine located 66 nucleotide upstream of the exon junction (Figure 3); the nomenclature refers to coding exons only.

**Figure 4:**
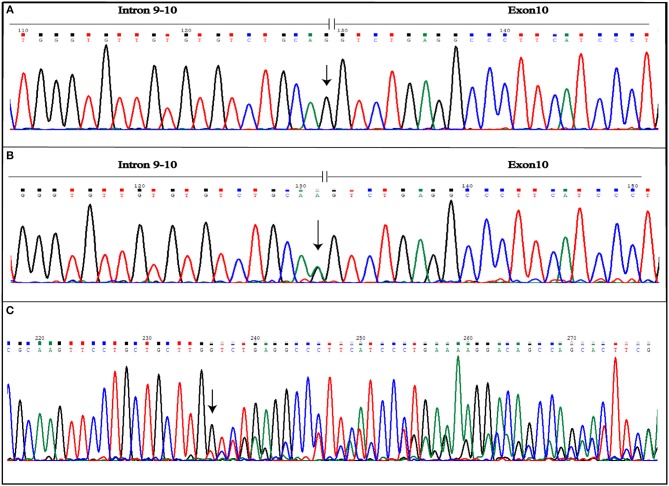
Novel IVS10-1G>A mutation detected in CCM2 gene. The figure shows both wild-type **(A)** and mutated **(B)** electropherograms. The heterozygous substitution is indicated by the arrow. The novel heterozygous variant causes splicing alteration that occurs including also the first nucleotide of exon 10, as shown by frameshift observed in electropherogram of the coding sequence **(C)**. The arrow indicates the point of the mutation.

More variants at the three CCM genes were, instead, detected in the second patient presenting a familial form. Results are summarized in Table 1. As shown, three single nucleotide polymorphisms (SNPs) were detected in *KRIT1* gene. Of these, rs17164451 and rs2027950 are intronic, while rs11542682is a synonymous substitution known to lead splicing impairment and, thus, associated to CCM lesion development (11). Another five SNPs were identified in the *CCM2* gene and, among these, c.358G>A (rs11552377) is reported in HGMD as associated to CCM development (12). Furthermore, a novel heterozygous intronic variant was detected and is located at the G nucleotide at the splice-acceptor site adjacent to the 10th exon. This is a single nucleotide substitution G>A that, according to the Human Genome Variation Society guidelines, was named IVS10-1G>A(c.1055-1G>A) (Figures 4A,B). Mutational analysis was also performed on proband's relatives and genotyping results are reported in Table 2.

**Table 1:** Variants detected in the 84-years old woman affected by familial CCM.

**Gene**	**Variant**
*KRIT1*	rs17164451 c.485+65G>C
	rs2027950 c.989+63C>G
	rs11542682 c.1980A>G p.Val660 = (HGMD MUT CM105502)
*CCM2*	rs2304689 c.205-36A>G
	rs11552377 (HGMD CM105503) c.358G>A p.Val120Ile
	rs55967204 c.745+98G>C
	rs2289367 c.915G>A p.Thr305 =
	rs2289369 c.915+119C>T
	IVS10-1G>A

**Table 2:** Intra-familial genotype-phenotype comparison.

	**Family members**									
	**VARIANT**	**I-2^†^**	**II-1^‡^**	**II-3^‡^**	**II-4^‡^**	**III-7**	**III-8**	**III-9**	**III-10**	**III-13^‡^**
Genotypes	rs17164451 c.485+65G>C	G/C	G/C	G/C	G/C	G/G	G/G	G/C	G/C	G/C
	rs2027950 c.989+63C>G	C/G	G/G	G/G	G/G	G/G	G/G	G/G	G/G	G/G
	rs11542682 c.1980A>G p.Val660 = (HGMD MUT CM105502)	A/G	A/G	A/G	A/G	A/A	A/A	A/G	A/G	A/G
	rs2304689 c.205-36A>G	A/G	A/G	A/G	A/G	A/A	A/G	A/G	A/A	A/G
	rs11552377 c.358G>A p.Val120Ile (HGMD CM105503)	G/A	G/G	G/G	G/G	G/G	G/G	G/G	G/G	G/G
	rs55967204 c.745+98G>C	G/C	G/G	G/G	G/G	G/G	G/G	G/G	G/G	G/G
	rs2289367 c.915G>A p.Thr305	G/A	G/A	G/A	G/A	G/G	G/A	G/A	G/G	G/A
	rs2289369 c.915+119C>T	C/T	C/T	C/T	C/T	C/C	C/T	C/T	C/C	C/T
	IVS10-1G>A	G/A	G/A	G/A	G/A	G/G	G/A	G/A	G/G	G/A
Phenotypes	Sex	F	M	M	F	F	F	F	F	F
	Age	84	60	58	48	34	32	31	29	30
	Age onset/diagnosis	64	60	50	48					30
	Number of lesions	4	>7	4	5					3
	Lesions localization	Pons Right parietal lobe	Brainstem cerebellar hemispheres	Brainstem	Brainstem, cerebral hemispheres	Negative MRI	Negative MRI	Negative MRI	Negative MRI	Posterior limb of the right internal capsule Left temporal lobe
	Symptoms	Epileptic seizures	Global transient amnesia	Asymptomatic	Headaches, Right hemiparesis, Hemi hyperesthesia					Asymptomatic

The table is divided in two sections. The upper shows allelic distribution among then family's members. The lower summarizes their main phenotypical features.

† Indicates the proband.

‡ Indicates the affected within the family. *Heterogeneity in lesions localization and in symptomatology are reported although equivalence of the genotypes*.

No large genomic rearrangements were detected by MLPA analysis.

### Characterization of novel variants and of mutated proteins features

Variants were found neither in HGMD, ExAC nor 1000G databases. As predicted, the novel variants detected in *KRIT1* and *CCM2* genes do not affect coding sequencing.

Hypothesis of normal splicing process alteration due to IVS15-66A>T variant was tested by sequencing of partial *KRIT1* cDNA. Analysis did not reveal any change in the nucleotide sequence, compared with the wild-type one (not shown). To date, therefore, its involvement in CCM development cannot be confirmed.

Aberrant splicing, instead, results as a consequence of IVS10-1G>A variant affecting the *CCM2* gene. The substitution causes loss of splicing acceptor site and the activation of an intronic cryptic acceptor site one nucleotide downstream of the wild-type, resulting in the deletion of the first nucleotide of exon 10 (c.1055delG) (Figure 4C). This leads to the change of amino acid at position 352 followed by a stop codon in position 353 (p.G352Vfs^*^2). Mutated protein is truncated by 92 amino acids, if compared with 444 amino acid wild-type ones.

### Population study

To estimate the frequency of *KRIT1* IVS15-66A>T and *CCM2* IVS10-1G>A variants in the population we selected two Italian and Greek control groups, each consisting of 150 healthy subjects. None of the controls carried the variants that, therefore, were defined as mutations.

**Figure 5:**
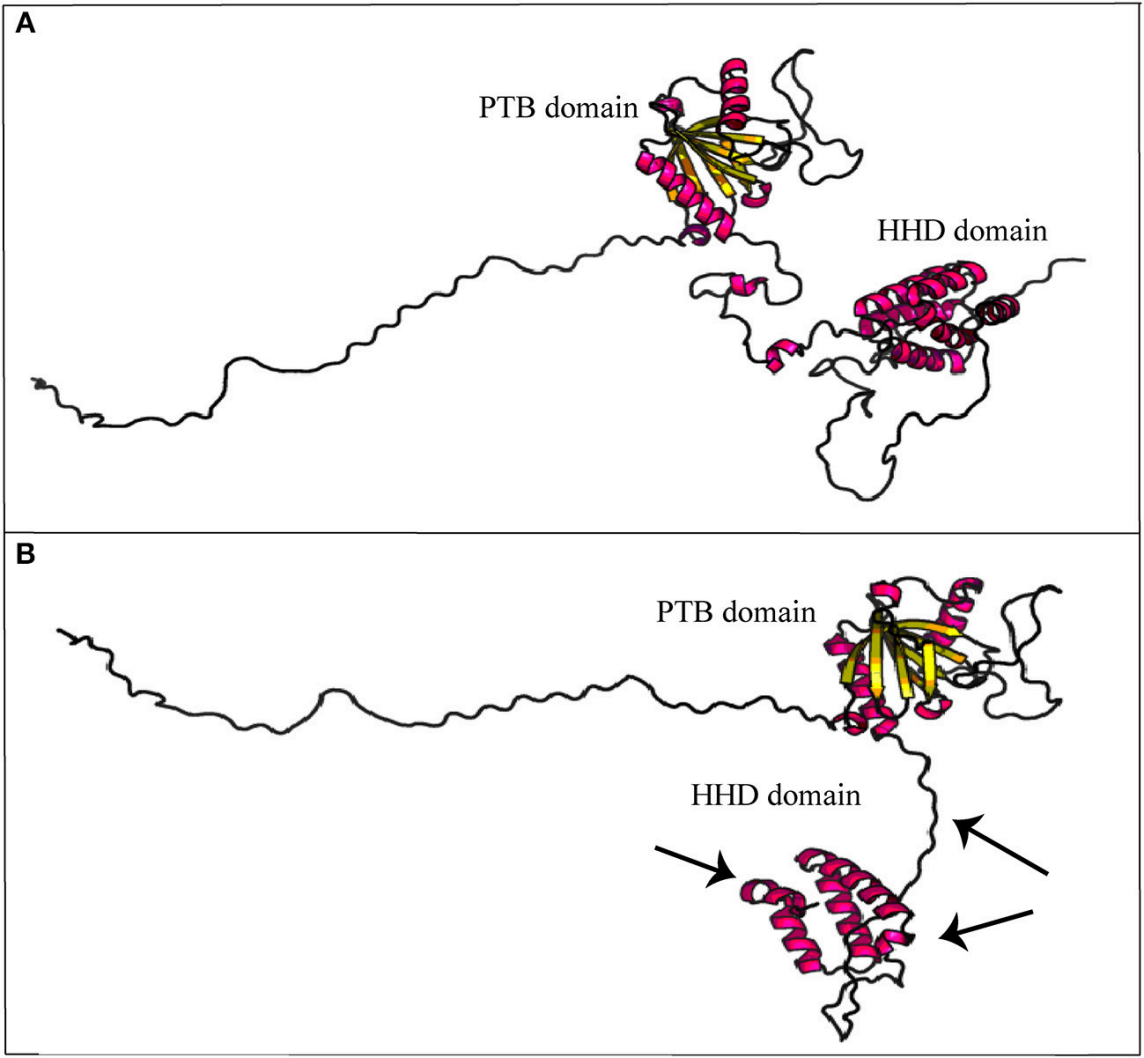
Secondary structure prediction of mutated malcavernin. The image compares native **(A)** and mutated **(B)** malcavernin secondary structures. As indicated by the arrows, the main structural modifications affect harmonin-homology domain (HHD) at C-terminus with consequent loss of two helices. Images were obtained by “Structure Prediction” module of RaptorX tool.

As described, *CCM2* IVS10-1G>A substitution impairs splicing of 10th exon leading to a 352 amino acid truncated protein. *In-silico* prediction of secondary structure performed at RaptorX tool shows how Harmonin Homology Domain (HHD) in C-terminus is partially disrupted (Figure 5).

## Discussion

Genetic bases of CCM are to date not well-defined, particularly for sporadic forms. Common features for these and hereditary cavernomatosis include neuro-radiological and clinically incomplete penetrance and variable expressivity (13). A low percentage of patients affected by the sporadic form of the disease, instead, carry germ-line mutations at one of the three CCM loci. Usually these patients show multiple lesions that, however, can remain asymptomatic during the course of life (14).

Conversely, familial forms tend to be symptomatic already in childhood (15).

The reason for this heterogeneity has not yet been elucidated. Different hypotheses have been proposed and among them, the existence of biallelic somatic mutations was proven by mutational analyses conducted on specimens derived from both inherited and sporadic lesions. Therefore, the Knudsonian two-hit mutation mechanism is one of the most accredited hypotheses to explain CCM pathogenesis (16). The cases here described report two affected patients with a not-completely overlapping clinical history. The first one is a man presenting a single CCM asymptomatic lesion that was discovered by chance. The patient carries the novel IVS15-66A>T germ-line mutation in the *KRIT1* gene that does not seem to compromise normal splicing, as shown by sequencing of the transcript. Despite its association with CCM development cannot be confirmed, also its total exclusion in pathogenesis cannot be considered correct. It may be due to a premature degradation of mutated transcript that did not allow its detection or an impairment of an alternative splicing form of the same transcript. None of these hypotheses have been demonstrated here. Moreover, the existence of somatic mutations cannot now be evaluated. Regarding the second case, this is an 84 year-old woman who developed many lesions which remained silent until the age of 60. The patient is affected by a familial form and more relatives are also affected. From genetic analysis, more variants associated to the onset of lesion were detected and, among these, the novel *CCM2* IVS10-1G>A. As reported, IVS10-1G>A causes the loss of a conventional splice site and gain of a cryptic one that includes the first nucleotide of exon 10. This results in a truncated protein, lacking 92 amino acids at C-terminus. In its structure, malcavernin contains a phosphotyrosine binding (PTB) domain by which interaction between krit1 and pdcd10 (17) is mediated, and a HHD (18) which interacts with TrkA inducing cell death (19), and with N-terminus of MEKK3 (20). CCM2-MEKK3 interaction is essential for maintaining endothelial barrier properties (21). Loss of this interaction causes an increased MEKK3 autophosphorylation resulting in an enhanced endothelial permeability due to Rho-/ROCK-dependent signaling perturbation (22). Therefore, CCM2–MEKK3 interaction needs negative regulation of MEKK3 activity (23).

In detail, Fisher et al. (18) proposed a crystallographic model of wild-type malcavernin in which HHD is formed by the S283-K375 amino acidic region folded to organize five α-helix motifs. In this region, amino acidic residuesP355-F356 are conserved and essential to stabilize the structure. As highlighted in Figure 5, the novel mutation here reported leads to the synthesis of a truncated and misfolded protein lacking in two helices of HHD. The consequence is an impairment in MEKK3 binding that triggers pathogenic signaling resulting in CCM phenotype.

Besides the complex molecular mechanisms that lead to lesion development, another important feature of CCM is the heterogeneity of the phenotypes. To date, only few data are available on genotype-phenotype correlation due to high clinical variability among patients. Variable expressivity is also observed within affected individuals belonging to the same family and linked to the same mutation. Together with allelic constitution, Table 2 reports phenotypical manifestations of the family here described. Despite lesion numbers tending to be homogeneous, they were distributed both at supratentorial and infratentorial regions as well as clinical manifestations which substantially differed among patients. Regarding genotypes, all affected individuals showed the same allelic assortment, with the exception of the proband (Table 2; I-2) who had a heterozygous condition for the two SNPs rs11552377 (c.358G>A p.Val120Ile) and rs55967204 (c.745+98G>C) in the *CCM2* gene. Only the carrier condition for rs11552377 could support the epileptic seizures that have arisen as the single clinical manifestation. As we previously described in a case-control study, indeed, this SNP is associated with both increased risk to develop CCM and a more severe symptomatology (12). An important data regards the segregation of the novel IVS10-1G>A mutation within the family. In Table 2, the proband is marked with a single asterisk while the affected relatives are twice starred. All examined 2nd generation members (Figure 2; II-1; II-3; II-4) were affected and manifested different symptoms although they carried the same variants. This is in accordance with the typical variable expressivity observed in CCM patients. Regarding the five members of the 3rd generation, only one (Figure 2; III-13) was positive at MRI scan. However, by comparison of their genotypes, the presence of the novel mutation IVS10-1G>A can be observed in three of them and, particularly, in the young affected woman (Figure 2; III-13) and in two of her healthy cousins (Figure 2; III-8 and III-9). This observation could be explained by two different hypotheses. The first is the lack of linkage between the pathological phenotype onset and the presence of the novel mutation. However, it produces deleterious effects on the malcavernin structure, therefore its involvement in CCM pathogenesis cannot be excluded. The second hypothesis, instead, regards the penetrance of the *CCM2* locus. Among CCM loci, *CCM2* is the only one reported with 100% of clinical and neuroradiological penetrance. However, in this case, seven members of the family carried the mutation but only five of them showed lesions at MRI scan. Therefore, for the first time, a penetrance for *CCM2* locus >100% is here reported and, to be precise, equivalent to 70%. Moreover, members II-1, II-3, II-4, III-9, and III-13 carried the same variants and, among these, III-9 is healthy. Based on the dynamic nature of familial CCM, if compared with 2nd generation members, absence of lesions in III-9 may be imputed to her younger age, and the probability that she will become illcannot be excluded. However, her coetaneous cousin already carries three lesions. As clearly evident by genotypes comparison, c.205-36A>G(rs2304689), c.915G>A (rs2289367), c.915+119C>T (rs2289369), and IVS10-1G>A in *CCM2* were in cis configuration, therefore other genetic or environmental factors influenced the phenotype. Moreover, the role of c.1980A>G p.Val660 = (rs11542682, HGMD MUT CM105502) SNP in *KRIT1*remains controversial. Despite being a synonymous substitution, it seems to impair splicing (11) and, therefore, it was associated to an increased risk of developing CCM. However, as in this case, it is frequently represented in families with hereditary CCM. The absence of the pathological phenotype in III-9 and III-10 despite them also harboring this variant could be attributed to not yet identified modifier factors that contribute to incomplete penetrance of CCM.

Among candidate loci that could act as modifier genes, surely *CCM2L* (C20orf160) can be considered. This is a *CCM2* homologous identified in zebrafish some years ago. Although knowledge about its functions is still poor and controversial, its involvement in cardiovascular development has been confirmed by several studies, as well as its participation in CCM protein signaling. Expression studies showed that *CCM2L* silencing in zebrafish embryos develop CCM-like phenotypes, comparable with knock-down *KRIT1* or *HEG1* ones. Like malcavernin, also ccm2l interacts with the N-terminus region of krit1 and, furthermore if one of these is deficient, it can be replaced by the other (24).

*CCM2L* expression was also observed in endothelial cells where, similar to *CCM2*, its C-terminus region binds N-terminus of MEKK3 (23) and, in particular, *CCM2L* can rescue *CCM2* deficiency. Therefore, in the case we presented, healthy *CCM2* IVS10-1G>A mutation carriers might not have developed CCM phenotype due to a greater compensatory activity of *CCM2L*. This hypothesis is being evaluated.

## Conclusions

In conclusion, here we describe two novel intronic mutations detected in *KRIT1* and in *CCM2* genes. Reported data are part of a wider project that aims to increase the spectrum of mutations associated to CCM development. In this context, we previously published data collected on more numerous cohorts (12, 25, 26). Regarding the novel mutations detected, we can affirm no evidence of association between *KRIT1* IVS15-66A>T, and therefore CCM development in a sporadic patient could not be a consequence of synthesis of an aberrant *KRIT1* transcript. Regarding IVS10-1G>A in *CCM2*, instead, it causes the formation of a truncated protein due to loss of the first nucleotide of the 10th exon that is deleted together with intron 9–10. This mutation was detected in several members belonging to a family affected by hereditary CCM. However, not all subjects harboring the mutation show lesions at MRI; this allows us to report, for the first time, a penetrance for *CCM2* locus lower than 100% and to enrich the spectrum of mutations responsible for CCM etiopathogenesis.

## Author contributions

CS designed the study and wrote the manuscript. LD carried out the genetic analysis and validation. ZK and SB performed the clinical evaluation and MRI examination of patients. RD participated in the validation of novel mutation and the sequence alignment, as well as drafting of manuscript. AS oversaw design and conceptualization of the study, and revising the manuscript.

### Conflict of interest statement

The authors declare that the research was conducted in the absence of any commercial or financial relationships that could be construed as a potential conflict of interest.

## Abbreviations

CCM: Cerebral cavernous malformations
ICH: Intracerebral hemorrhage
MRI: Magnetic Resonance Imaging,
Krit1: Krev interaction trapped 1
Pdcd10: Programmed cell death protein 10
ECM: cell-extra cellular matrix
HGMD: Human Gene Mutation Database
MLPA: Multiplex Ligation-Dependent Probe Amplification
PCR-RFLP: Polymerase Chain Reaction- Restriction Fragment Length Polymorphism
SNPs: single nucleotide polymorphisms
PTB: phosphotyrosine binding
HHD: Harmonin Homology Domain
MEKK3: mitogen-activated protein 3 kinase.
